# Bench-to-Bedside Insights into the Challenges of Immunosuppression in Sepsis

**DOI:** 10.3390/pathogens15020159

**Published:** 2026-02-02

**Authors:** Shaowen Huang, Siyuan Huang, Xiaofei Huang, Xifeng Feng, Rui Wang, Di Liu, Jianhui Sun, Huacai Zhang, Juan Du, Li Lin, Qinyuan Li, Anyong Yu, Ling Zeng

**Affiliations:** 1 Department of Trauma Medical Center, State Key Laboratory of Trauma and Chemical Poisoning, Daping Hospital, Army Medical University, Chongqing 400042, China; 13679544208@139.com (S.H.); syhuang@tmmu.edu.cn (S.H.); zyjzhxf@zmu.edu.cn (X.H.); fengxifeng@tmmu.edu.cn (X.F.); wangr5767@tmmu.edu.cn (R.W.); heidi678@tmmu.edu.cn (D.L.); sendy@tmmu.edu.cn (J.S.); jinx223875@tmmu.edu.cn (H.Z.); dujuan1982@tmmu.edu.cn (J.D.); 2 Department of Emergency, Affiliated Hospital of Zunyi Medical University, Zunyi 563003, China; 3 Unit 94654 of the Chinese People’s Liberation Army, Nanjing 210046, China; lovely-l2@163.com; 4 Department of Respiratory Medicine, National Clinical Research Centre for Child Health and Disorders, Ministry of Education Key Laboratory of Child Development and Disorders, Chongqing Key Laboratory of Child Rare Diseases in Infection and Immunity, China International Science and Technology Cooperation Base of Child Development and Critical Disorders, Children’s Hospital of Chongqing Medical University, Chongqing 400014, China; liqinyuan@hospital.cqmu.edu.cn

**Keywords:** sepsis, immunosuppression, immune monitoring, biomarkers, immunomodulatory therapy

## Abstract

Sepsis remains a leading cause of global mortality and is characterized by a dysregulated host immune response to infection. Early deaths often result from hyperinflammation and organ dysfunction, whereas late-stage mortality is increasingly attributed to sepsis-induced immunosuppression, leading to secondary infections and viral reactivation. Challenges persist in the identification and management of sepsis-induced immunosuppression, including the lack of standardized immune monitoring methods, the absence of reliable immune biomarkers to guide therapy, and the limited success of immunomodulatory therapies in clinical trials. This review comprehensively summarizes the pathophysiology of sepsis-induced immunosuppression, encompassing immune cell apoptosis and exhaustion, the expansion and activation of immunomodulatory cells, metabolic reprogramming, epigenetic alterations, and iatrogenic factors. We also discuss current diagnostic challenges and explore emerging immunomodulatory strategies, such as cytokine therapies, immune checkpoint inhibitors, and metabolic modulators, as potential approaches to restore immune function. Finally, we highlight the importance of immune phenotyping and individualized precision medicine in the future management of sepsis, and integrating multidisciplinary approaches from mechanistic research to targeted therapies holds promise for improving patient outcomes.

## 1. Introduction

Sepsis is defined as life-threatening organ dysfunction caused by a dysregulated host response to infection. Its incidence and mortality are high worldwide [1]. According to global statistics from 2017, nearly 50 million annual cases of sepsis and more than 10 million sepsis-related deaths occurred, accounting for approximately one-fifth of all global deaths and representing one of the most significant burdens on health care expenditure [2]. Consequently, the World Health Organization (WHO) declared sepsis a global health priority in 2017 [3].

In China, the incidence of sepsis is also increasing annually. Statistics from 2017 to 2019 indicate that the incidence increased from 328.25 to 421.85 per 100,000 people and that the annual number of sepsis-related deaths was close to 1 million, with adults over 65 years old accounting for approximately 57.5% of the deaths [4]. In the United States, 75% of sepsis-related deaths occur in patients aged 65 and older [5], with pneumonia being the leading precipitating cause [6]. However, the pathogen detection rate for pneumonia is low, with an etiology identified in only 38% of community-acquired pneumonia (CAP) patients; among these, viruses account for approximately 40% [7]. Trauma is a significant trigger of sepsis in individuals under 45 years of age; severe trauma and blood loss markedly increase the risk of sepsis and multiple organ failure [8].

**Figure 1:**
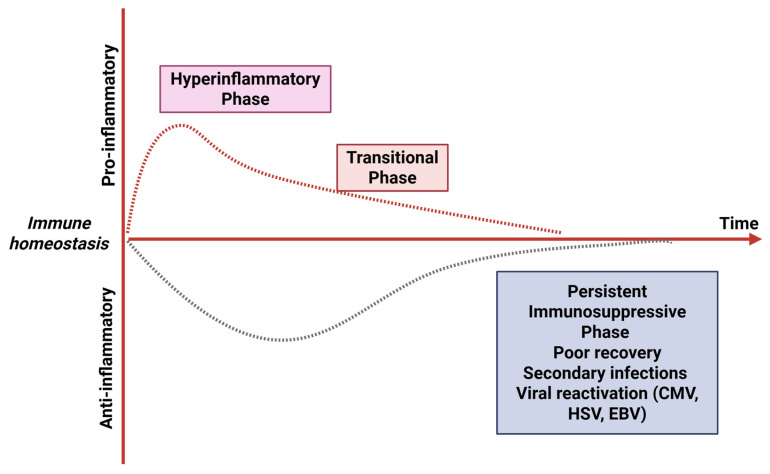
**Timeline of immunosuppression.** In the initial stage of sepsis, the body undergoes metabolic reprogramming and epigenetic modifications in immune cells to eliminate pathogens, during this phase, immune cell metabolism is markedly enhanced, leading to the release of a large number of pro-inflammatory cytokines, resulting in a predominantly pro-inflammatory response. As the disease progresses, the body gradually enhances the anti-inflammatory response to balance systemic inflammation and protect organs and tissues, while the pro-inflammatory response gradually weakens. This transitional phase is characterized by the coexistence of pro-inflammatory and anti-inflammatory processes. Ultimately, upon pathogen clearance or exhaustion of immune cells, the pro-inflammatory response subsides. However, due to the failure to promptly replenish and restore immune cell numbers and function, along with the expansion of immunosuppressive cells, the patient enters an immunosuppressive phase. During this stage, patients become highly susceptible to secondary infections and viral reactivation. CMV, cytomegalovirus; HSV, herpes simplex virus; EBV, Epstein–Barr virus.

From the traditional perspective, sepsis is viewed as a hyperinflammatory response. However, a growing number of studies have indicated that immunosuppression plays a critical role in the pathogenesis, progression, and outcomes of sepsis [9], which is characterized by extensive immune cell apoptosis, expansion of immunosuppressive cell populations, elevated levels of anti-inflammatory cytokines, and upregulated expression of immune checkpoints [10], predisposing patients to secondary infections and viral reactivation (such as cytomegalovirus, herpes simplex virus, and Epstein–Barr virus), which is closely associated with increased long-term mortality [11] (Figure 1).

In summary, immunosuppression is a central determinant of sepsis prognosis and is associated with high mortality, with approximately 30% of patients dying within the following year. Furthermore, about 40% of survivors are rehospitalized within 90 days of discharge, and one-sixth suffer from long-term disability [12]. This collective burden of sequelae is also referred to as post-sepsis syndrome (PSS) [13]. Therefore, the profound clinical impact of sepsis-induced immunosuppression motivates us to summarize its mechanisms, diagnostics, and therapeutic strategies from the bench to the bedside.

## 2. Bench: Cellular and Molecular Mechanisms of Immunosuppression in Sepsis

The mechanisms of immunosuppression in sepsis are complex and have not yet been fully elucidated. Currently recognized key mechanisms include dysregulation of the innate immune system, exhaustion of the adaptive immune system, metabolic reprogramming, epigenetic alterations, and iatrogenic factors. In this study, we describe the principal mechanisms contributing to immunosuppression in sepsis.

Under physiological conditions, the host immune system maintains homeostasis. During pathogen invasion, innate immune cells recognize pathogen-associated molecular patterns (PAMPs) and damage-associated molecular patterns (DAMPs) via pattern recognition receptors (PRRs), initiating a local inflammatory response to eliminate pathogens. This response is self-limiting, and the immune system returns to homeostasis after pathogen clearance. However, a dysregulated host response to infection can disrupt this balance, leading to systemic inflammation and ultimately triggering sepsis [14]. In the early stage of sepsis, the host rapidly recognizes and clears pathogens through complement, antibodies, neutrophils, monocytes, and macrophages, releasing cytokines such as interleukin-6 (IL-6), interleukin-1 beta (IL-1β) and tumor necrosis factor α (TNF-α) and forming a positive feedback loop that can trigger a cytokine storm [11,15].

**Figure 2:**
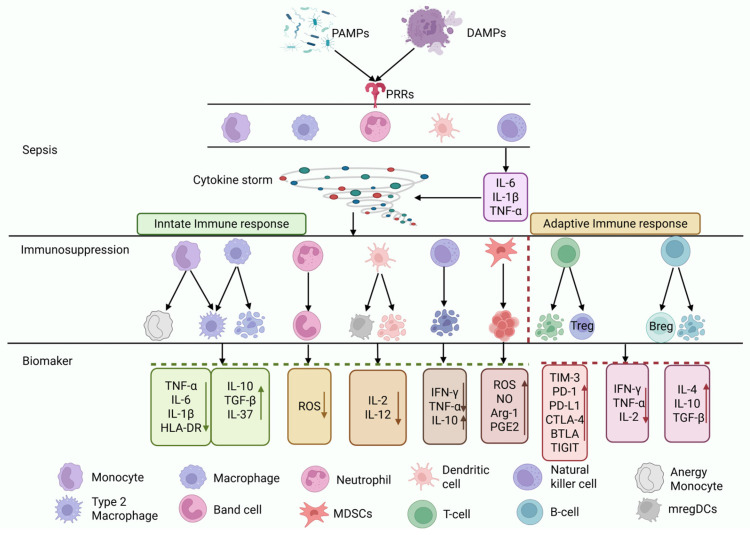
**Immunosuppression mechanisms.** In the early stages of sepsis, PAMPs and DAMPs are recognized by PRRs, prompting immune cells of the innate immune system to release proinflammatory cytokines such as IL-6, IL-1β, and TNF-α. These cytokines continuously amplify the inflammatory response, ultimately leading to a cytokine storm. This storm causes multiorgan dysfunction, including damage to immune cells themselves. To counterbalance the immune response, the immune microenvironment begins to shift toward an anti-inflammatory state. Immune cells undergo apoptosis or become dysfunctional, differentiating into anti-inflammatory or anergic phenotypes. The release of anti-inflammatory cytokines increases, ultimately resulting in immunosuppression. TNF-α, tumor necrosis factor-alpha; IL, interleukin; HLA-DR, human leukocyte antigen-DR isotype; TGF-β, transforming growth factor-beta; ROS, reactive oxygen species; IFN-γ, interferon-gamma; NO, nitric oxide; Arg-1, arginase-1; PGE2, prostaglandin E2; TIM-3, T cell immunoglobulin and mucin-domain containing-3; PD-1, programmed cell death protein 1; PD-L1, programmed cell death ligand 1; CTLA-4, cytotoxic T-lymphocyte-associated protein 4; BTLA, B- and T-lymphocyte attenuator; TIGIT, T cell immunoreceptor with Ig and ITIM domains. Black arrows show disease progression, case transition, and outcomes. Colored arrows show biomarker changes (upward represent increase, downward represent decrease). Vertical red dashed lines are separators, whereas the horizontal red and green bars represent the adaptive and innate immune cell groups, respectively.

As the condition progresses, anti-inflammatory mechanisms are activated to curb excessive inflammation, and this may lead to immunosuppression, manifested by the following: reduced neutrophil chemotaxis and oxidative burst capacity, along with increased programmed cell death ligand 1 (PD-L1) expression and the release of interleukin-10 (IL-10), resulting in an immunosuppressive phenotype [3]; increased monocyte apoptosis that can significantly decrease human leukocyte antigen-DR isotype (HLA-DR) expression, accompanied by increased release of anti-inflammatory factors such as IL-10 and transforming growth factor-beta (TGF-β) and reduced pro-inflammatory factors such as TNF-α; decreased dendritic cell (DC) numbers and functional impairment, leading to weakened T cell responsiveness to infection [16]; marked expansion of myeloid-derived suppressor cells (MDSCs) and regulatory T cells (Tregs); increased T cell apoptosis and functional exhaustion as indicated by elevated expression of programmed cell death protein 1 (PD-1), T cell immunoglobulin and mucin-domain containing-3 (TIM-3), and cytotoxic T-lymphocyte-associated protein 4 (CTLA-4); reduced B-cell counts and a shift toward regulatory B cells (Bregs), with diminished antibody production capacity; and significant reduction in the numbers and functional suppression of natural killer cells (NK) and natural killer T cells (NKT), along with a sharp decline in cytokine production, particularly interferon-gamma (IFN-γ), thus increasing susceptibility to secondary infections, viral reactivation, and long-term immune deficiency [9] (Figure 2).

### 2.1. Innate Immune System Dysregulation

#### 2.1.1. Monocytes/Macrophages

Monocytes are a critical component of the innate immune system and serve as a versatile reserve force in immune regulation. They are a highly heterogeneous population of innate immune cells with independent functions that are capable of differentiating into various cell types under different physiological and pathological conditions. These include tissue-resident macrophages, inflammatory effector cells, antigen-presenting cells, and regulatory cells [17]. Recent research by Keramati et al. [18] demonstrated that during acute inflammation in humans, monocytes can differentiate into a subset termed “infMonos”, which can suppress T cell proliferation and cytokine secretion; these infMonos contribute to immunosuppression by impairing the type I interferon (IFN-I) signaling pathway, disrupting myeloid cell maturation, and decreasing monocyte responsiveness to various pathogenic stimuli. Furthermore, functional exhaustion of monocytes leads to reduced HLA-DR expression, which is a key mechanism and reliable biomarker of sepsis-induced immunosuppression, as well as a critical feature of monocyte paralysis [9].

Macrophages are at the center of the innate immune system, exhibit high plasticity and can polarize into distinct functional phenotypes in different microenvironments. In the proinflammatory stage of sepsis, they may adopt the M1 phenotype, which secretes proinflammatory cytokines such as TNF-α, IL-6, and IL-1β, promoting immune activation and clearance of invading pathogens. In contrast, during the immunosuppressive phase, polarization tends to shift toward the M2 phenotype, which promotes the secretion of IL-10 and TGF-β, leading to immune paralysis [19]. IL-10 can further suppress the activation of macrophages, DCs, and T cells, thus promoting the expansion of Tregs and exacerbating immunosuppression [20]. Notably, CD169+ macrophages represent a major source of IL-10 in sepsis. In LPS-induced septic mice model, CD169+ macrophages produce substantial amounts of IL-10 during early infection, directly contributing to the induction of immunosuppression [21].

#### 2.1.2. Neutrophils

Neutrophils serve as antimicrobial sentinels in the human immune defense system. Under homeostatic conditions, they sense chemokine gradients and migrate directionally to the site of infection, where they phagocytose pathogens and eliminate them through the formation of phagosomes, releasing reactive oxygen species (ROS) and granule enzymes, or by extruding neutrophil extracellular traps (NETs) that are composed of DNA, histones, and granular proteins to capture and kill microbes. Additionally, neutrophils interact with DCs and T cells, bridging innate and adaptive immunity [22]. In severe infections such as sepsis, despite a marked increase in peripheral neutrophil counts, patients remain susceptible to infections. An important reason is the impaired function of neutrophils mentioned above [22]. Moreover, severe inflammation prompts the bone marrow to release immature neutrophil subsets, such as the CD200Rhigh subset identified by Kim et al. [23]. In septic mice, this subset highly expresses insulin-like growth factor 1 (IGF-1), which promotes the expansion of Tregs, while Treg-derived TGF-β subsequently further suppresses immune responses. Furthermore, the CXCR2^+^ neutrophil subset that was identified by Shen et al. [24] in bronchoalveolar lavage fluid (BALF) from septic patients has also been implicated in immunosuppression during sepsis. In summary, during sepsis, neutrophils act not only as failed executors of bacterial clearance but also as active participants in immune paralysis.

#### 2.1.3. Dendritic Cells

As pivotal antigen-presenting cells, DCs serve as a crucial bridge between innate and adaptive immunity. A reduction in their numbers and impairment of their function compromise T cell responsiveness to infection [16], leading to a diminished capacity to activate T cells and promoting the induction of T cell exhaustion. Decreases in DC number and function significantly contribute to the development of sepsis-induced immunosuppression and are closely associated with secondary infections and mortality [25].

During sepsis, DCs can undergo functional exhaustion and differentiate into specialized subsets that promote immune tolerance. A key example is “mature DCs enriched in immunoregulatory molecules” (mregDCs), which were first identified in a murine sepsis model and later found to have a conserved counterpart (Lysosomal-associated membrane protein 3 + DCs (LAMP3 + DCs)) in the bronchoalveolar lavage fluid of patients with severe COVID-19, a condition associated with sepsis. In mice, mregDCs increase CD4+ T cell proliferation and IL-2/IL-12 secretion while skewing their differentiation toward the Treg and Th2 lineages, thereby fostering immunosuppression [26]. This functional impairment and phenotypic shift in DCs significantly contribute to the compromised host defenses in sepsis.

#### 2.1.4. NK Cells

NK cells are a subset of lymphocytes that serve as a bridge between the innate and adaptive immune systems and exhibit features of immunological memory [27]. They represent a crucial component of the immune system. NK cells secrete a variety of cytokines and chemokines, such as TNF-α, IFN-γ, and IL-10, that modulate the immune activity and function of other immune cells. Through interactions with antigen-presenting cells such as DCs, T and B cells are activated, resulting in the formation of an integrated immune network that promotes systemic immune activation and strengthens antitumor and anti-infection responses [28].

In sepsis, NK cells exhibit increased PD-L1 expression, reduced cell numbers, functional suppression, and a marked decrease in the production of cytokines, particularly IFN-γ [9,25]. In summary, dysregulation of NK-cell number and function is closely associated with the development of immunosuppression in sepsis.

#### 2.1.5. Myeloid-Derived Suppressor Cells

The expansion of MDSCs is a hallmark of immunosuppression in sepsis, with T cells being their primary target [29]. MDSCs are a heterogeneous population of immature myeloid cells originating from the bone marrow that acquire potent immunosuppressive capabilities under pathological conditions such as cancer and infection [30,31]. They also perform immunosuppressive functions through multiple mechanisms, including the production of ROS, nitric oxide (NO), arginase-1 (Arg-1), and prostaglandin E2 (PGE_2_). These mediators promote the expansion of Tregs and inhibit T cell proliferation and function [32]. MDSCs also suppress the activity of B cells and NK cells [33].

In the context of cancer, MDSCs directly inhibit T cell activity through the expression of immune checkpoint molecules such as PD-L1, CTLA-4, galectin-9 (Gal-9), and CD155. They also upregulate the expression of enzymes such as Arg1, indoleamine 2,3-dioxygenase (IDO), and cationic amino acid transporter-2B (CAT-2B), which deplete essential amino acids such as arginine, tryptophan, and cysteine, thereby impairing T cell proliferation and function [31].

Similarly, during infections, including sepsis and bacterial and viral infections, persistent expansion and accumulation of MDSCs suppress immune responses, leading to protracted infection and aggravated immunosuppression. Interestingly, MDSCs may play a protective role in the early stages of infection [13,25,34]. Furthermore, MDSCs are considered central drivers of immunosuppression in persistent inflammation and catabolism syndrome (PICS) and are closely associated with prolonged ICU stays, recurrent infections, and long-term mortality in septic patients [35].

### 2.2. Exhaustion of the Adaptive Immune System

Exhaustion of the adaptive immune system is closely linked to the development of immunosuppression in sepsis. The number and function of lymphocytes directly reflect and determine the state of adaptive immunity. In sepsis, lymphopenia results from both increased apoptosis and impaired proliferation [36], with apoptosis being the predominant mechanism [37].

#### 2.2.1. Lymphocyte Apoptosis

A key event in sepsis-induced immunosuppression is the extensive apoptosis of T cells, B cells, and DCs. Autophagy impairment in T cells during sepsis promotes apoptosis, and the apoptotic cells themselves exert immunosuppressive effects, further dampening immune responses [9]. Apoptotic cells suppress proinflammatory cytokine production in macrophages, promote the release of anti-inflammatory factors such as IL-10 and TGF-β, skew immunity toward a Th2 response while suppressing Th1 responses, and induce the expansion of Tregs, collectively contributing to a marked reduction in CD4+ T cells [38]. A study by Long et al. [39]. demonstrated in a mouse model that during acute systemic inflammatory responses such as sepsis, stimulator of interferon genes (STING) activation induces massive CD4+ T cell apoptosis, exacerbating immunosuppression and worsening outcomes. In contrast, Notch signaling protects CD4+ T cells by suppressing the STING pathway, suggesting that Notch activation or STING inhibition may represent potential immunoprotective strategies. Notably, the extensive apoptosis of mature lymphocytes, including both B and T cells, leads to the selective depletion of memory B cells and the loss of immunological memory [40].

Sepsis results in reduced numbers of both CD4+ and CD8+ T cells [13], but its impact extends beyond mere quantitative loss. Although lymphocyte counts may recover to levels comparable to those of healthy individuals within six months after hospital discharge [41], functional impairments persist: CD4+ T cells exhibit impaired immune responses to ex vivo stimulation with Aspergillus antigens, and memory CD8+ T cells show reduced antigen sensitivity [13]. The combined loss of immune cells and their long-term functional deficiencies are critical factors underlying the increased susceptibility to secondary infections and poor long-term outcomes in sepsis patients.

#### 2.2.2. T Cell Exhaustion

T cell exhaustion is characterized by a reduced proliferative capacity, decreased cytokine production, impaired cytotoxicity, and the overexpression of a distinct set of inhibitory receptors, such as PD-1 and TIM-3 [42].

Increased PD-1 expression on T cells is associated with impaired T cell proliferation, an increased incidence of nosocomial infections, and increased mortality in sepsis patients. This occurs because the binding of PD-1 to its ligands on exhausted T cells triggers the release of immunosuppressive molecules and ultimately leads to immune cell death [43]. TIM-3 exerts its inhibitory function by interacting with multiple ligands, including Gal-9, phosphatidylserine, high mobility group box 1 (HMGB1), and carcinoembryonic antigen-related cell adhesion molecule 1 (CEACAM-1) [44]. A study demonstrated that the binding of TIM-3 to HMGB1 suppresses the NF-κB signaling pathway, leading to decreased production of inflammatory cytokines and inhibition of T cell function, and inhibition of TIM-3 expression reduces mortality in mice with sepsis-induced immunosuppression [45]. These findings highlight the critical role of elevated immune checkpoint expression in T cell exhaustion and identify them as potential therapeutic targets for sepsis-associated immunosuppression.

#### 2.2.3. Tregs

Tregs represent a distinct subset of immune cells, accounting for approximately 5–10% of CD4+ T cells [46]. Under homeostatic conditions, Tregs help maintain self-tolerance by suppressing effector T cell function, leading to decreased activity of the latter. They also exert inhibitory effects on monocytes and neutrophils [14]. However, the proportion of Tregs can vary under different immune conditions. For example, during the early hyperinflammatory phase of sepsis, a decrease in their frequency is associated with excessive inflammatory responses [47]. In contrast, in the later immunosuppressive phase, although the overall number of CD4+ T cells is reduced, the relative increase in the proportion of Tregs is linked to immunosuppression and long-term mortality in septic patients [48].

The mechanisms by which Tregs contribute to immunosuppression have not yet been fully elucidated, but several key pathways have been identified: the release of anti-inflammatory cytokines such as TGF-β and IL-10; the upregulation of inhibitory costimulatory receptors on effector immune cells, including TIM-3, PD-1, T cell immunoreceptor with Ig and ITIM domains (TIGIT), CTLA-4 and neuropilin-1; epigenetic modifications of the *Foxp3* gene, which increase Treg stability during lymphopenic conditions; and a metabolic shift from glycolysis toward oxidative phosphorylation, which strengthens their suppressive capacity [46]. Therefore, the modulation of Treg function may represent a potential therapeutic strategy for ameliorating sepsis-induced immunosuppression.

### 2.3. Metabolic Reprogramming

Oxidative phosphorylation (OXPHOS) serves as the primary energy source for resting cells. This occurs within the mitochondria and produces ATP with high efficiency (approximately 36 ATP molecules per glucose molecule). In contrast, glycolysis takes place in the cytoplasm, where one molecule of glucose is broken down into two molecules of pyruvate, resulting in a net gain of two ATP molecules. Although less efficient at ATP production, this pathway is oxygen independent and can be rapidly activated. This phenomenon, in which cells preferentially utilize glycolysis even in the presence of adequate oxygen, is termed the Warburg effect [9].

**Figure 3:**
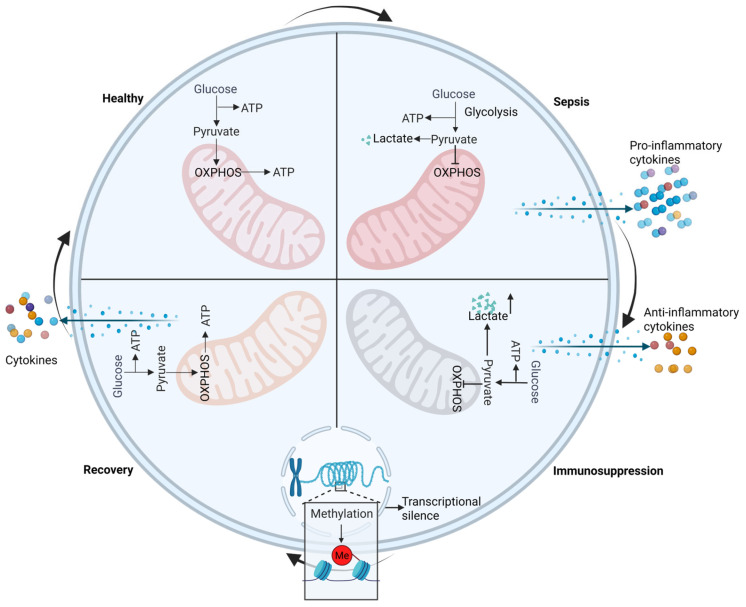
**Metabolic reprogramming and epigenetic alterations.** Under healthy conditions, immune cells primarily rely on oxidative phosphorylation (OXPHOS) for metabolism. The ATP required for immune responses is produced mainly within mitochondria—a process that is aerobic and highly efficient. However, during sepsis, the energy metabolism of immune cells shifts from efficient OXPHOS to rapid glycolysis. This switch allows rapid energy generation to fuel the proinflammatory response and rapidly eliminate pathogens. Nevertheless, this metabolic mode is inefficient in terms of glucose utilization and, when sustained, leads to lactate accumulation, resulting in cellular apoptosis or dysfunction. Consequently, the production of proinflammatory cytokines decreases, while that of anti-inflammatory cytokines increases. Additionally, epigenetic modifications occur, characterized by increased DNA methylation, which silences genes involved in the proinflammatory response, driving the system into an immunosuppressive phase. Upon patient recovery, the cells gradually revert to a healthy state, and their metabolic profile slowly shifts back to being dominated by OXPHOS. However, methylation patterns may persist for an extended period and can be inherited by daughter cells, potentially contributing to long-term immunosuppression. The extracellular arrows represent changes in the disease state of sepsis.

In the early stages of sepsis, immune cells shift their metabolism from efficient OXPHOS to rapid glycolysis to meet energy demands, facilitating pathogen clearance [49,50]. However, prolonged glycolysis leads to lactate accumulation, inhibits M1 macrophage polarization while promoting the M2 phenotype, impairs T cell migration and function, and suppresses inflammatory gene expression through mechanisms such as GPR81 signaling and histone lactylation. These changes significantly contribute to the immunosuppressive characteristics of late sepsis [49] (Figure 3).

Studies have indicated that the mTOR–HIF-1α pathway acts as a central regulator of immunometabolism and that its activity can be enhanced by IFN-γ treatment [51]. Similar metabolic effects have been observed with fructose-1,6-bisphosphate and AMP-activated protein kinase (AMPK)/mechanistic target of rapamycin (mTOR) regulators [50]. Furthermore, Luo et al. [52] reported that celastrol (Cel), a natural bioactive compound derived from the traditional Chinese herb Tripterygium wilfordii, has anti-inflammatory and immunomodulatory effects. This compound has been shown to suppress the inflammatory response and Warburg effect in macrophages by inhibiting the PKM2 signaling pathway, thereby restoring mitochondrial function, as indicated by an increased oxygen consumption rate (OCR), and ultimately protecting mice from sepsis and lethal endotoxemia.

#### Activation of the Tryptophan Metabolic Pathway

Tryptophan and its metabolites play a significant regulatory role in balancing immune responses [53]. IDO is the key enzyme responsible for converting tryptophan into kynurenine [31]. Tryptophan degradation via the IDO pathway and the subsequent production of kynurenine are recognized as drivers of immunosuppression in sepsis and are associated with increased T cell apoptosis [54]. In addition, IDO activation contributes to immune suppression through the inhibition of NK cells and the recruitment and activation of Tregs and MDSCs [31].

Tattevin et al. [55] reported that increased IDO activity in patients with severe sepsis and septic shock is correlated with mortality, suggesting that IDO production may help characterize monocyte reprogramming in sepsis. Another study confirmed that IL-6 promotes skeletal muscle catabolism during abdominal infection-induced sepsis by activating the tryptophan–indoleamine 2,3-dioxygenase 1–kynurenine pathway [56]. In an observational study, Riché et al. [53] reported that patients with abdominal septic shock exhibited elevated IDO levels for a long time. Therefore, IDO represents a potential therapeutic target for reducing tryptophan catabolic breakdown and alleviating immunosuppression in sepsis.

### 2.4. Epigenetic Regulation

Epigenetic alterations in immune cells play a key regulatory role in sepsis-related immunosuppression. Throughout the course of sepsis, widespread changes in the gene expression profiles of immune cells, characterized by downregulation of proinflammatory genes and upregulation of anti-inflammatory genes, including those marked by repressive histone modifications such as H3K9me2 and H3K27me3, occur. These changes can even affect bone marrow progenitor cells, contributing to long-term immune dysfunction [43] (Figure 3).

Among these, DNA methylation is closely associated with immunosuppressive phenotypes, such as increased IL-10 production, impaired T cell function, and reduced antigen-presentation capacity [57]. Caldwell et al. [58] demonstrated that in severe infections, including sepsis, DNA methylation patterns in bone marrow monocytes are closely linked to monocyte functional exhaustion and the establishment of immune memory. Specifically, increased methylation is generally associated with gene silencing, whereas decreased methylation is correlated with increased gene expression. Similar conclusions were drawn by Godoy-Tena et al. [59] in a study of COVID-19-associated sepsis.

Notably, the epigenetic state of innate immune cells can vary significantly depending on the priming stimulus. For example, LPS can induce immune tolerance, whereas β-glucan promotes trained immunity and can even reverse LPS-induced tolerance [60]. Another study revealed that hospital-acquired pneumonia (HAP) induces epigenetic reprogramming in alveolar macrophages. The key regulator signal-regulatory protein α (SIRPα) suppresses phagocytic function and promotes an immunosuppressive microenvironment, leading to long-term impairment of cellular function [61].

In summary, although aberrant epigenetic modifications can be heritable and cause long-term immune dysregulation, their reversible nature makes them a promising novel strategy for reversing immune tolerance.

### 2.5. Iatrogenic Factors

Glucocorticoids possess potent anti-inflammatory effects and have been used as an adjunctive therapy for septic shock for more than four decades, with a favorable safety profile [62]. Several large-scale trials have reported that compared with control treatment, treatment with glucocorticoids can shorten the time to shock reversal, duration of first mechanical ventilation, ICU length of stay, and transfusion requirements in patients with septic shock without significantly increasing the incidence of secondary infections [62,63]. However, most studies were designed with 90-day endpoints, and immunosuppression often persists long beyond this period in sepsis patients [54]. Therefore, whether glucocorticoid use leads to more profound or prolonged immunosuppression after 90 days remains unclear.

Norepinephrine (NE) represents the first-line vasopressor for managing septic shock because of its ability to stabilize hemodynamics. However, Stolk et al. [64] demonstrated that NE exerts notable immunosuppressive effects through activation of the β_2_-adrenergic receptor (β_2_-AR) and protein kinase A (PKA) signaling pathways, and NE suppresses immune cell metabolism, including both glycolysis and OXPHOS; inhibits the release of proinflammatory mediators such as TNF-α, IFN-γ–induced protein 10 (IP-10), and IL-1β; enhances the expression of the anti-inflammatory cytokine IL-10; and reduces ROS production. These changes collectively impair the bactericidal capacity of leukocytes and may exacerbate sepsis-associated immunoparalysis. In contrast, vasopressin does not have such immunomodulatory activity [64]. Therefore, norepinephrine represents a significant and modifiable driver of immunosuppression, warranting further investigation into alternative vasopressors or combination strategies with β-blockers to mitigate these effects [65].

Notably, a growing body of evidence indicates that longer courses of antimicrobial therapy are not superior to shorter courses in sepsis [66]. Conversely, inappropriate antibiotic use during sepsis, such as the excessive or prolonged use of broad-spectrum agents, may be a potential contributor to sepsis-induced immunosuppression by inducing gut microbiota dysbiosis, increasing the risk of bacterial translocation, promoting an immunosuppressive state, and increasing the risk of secondary infections and mortality. Furthermore, inappropriate antibiotic use is closely associated with organ dysfunction, such as acute kidney injury and muscle wasting [67]. Consequently, precise and rational antibiotic stewardship is imperative.

Opioids, such as fentanyl and morphine, are widely used in the ICU for sedation and analgesia. However, long-term use of opioids may be associated with serious side effects, including immunosuppression. Remifentanil can significantly inhibit the activation of neutrophils and suppress the activity of NK cells, while morphine can inhibit the phagocytic and bactericidal activities of macrophages, impair antigen presentation by dendritic cells, compromise T lymphocyte function, and suppress T cell proliferation [68]. A retrospective study on cancer patients undergoing chemotherapy who are at risk for developing febrile neutropenia and infections found that opioid use increased the probability of infection by 7.13 times and raised the probability of death/hospice care within 30 days after discharge by 2.30 times [69]. Peng et al. [70] found that morphine activates NOD-like receptor X1 (NLRX1) in microglia, impairing lysosomal function and leading to insufficient mitochondrial uptake in microglia, ultimately resulting in host immunosuppression and increased susceptibility to brain diseases. Interestingly, the effects of opioids on the immune system may be complex, context-dependent, and drug-specific. For example, Chemello et al. [71] demonstrated that fentanyl upregulates the mRNA levels of pro-inflammatory mediators in primary rat microglia and human monocyte-derived macrophages (MDMs), enhances LPS-induced secretion of pro-inflammatory mediators, and activates the NF-κB signaling pathway in MDMs and HEK293 cells stably transfected with human Toll-like receptor 4 (TLR4), Myeloid differentiation factor 2 (MD-2), and CD14 genes (HEK-Blue hTLR4 cells).

## 3. Bridging the Gap: Assessment of the Immunosuppressive State and Biomarkers

**Figure 4:**
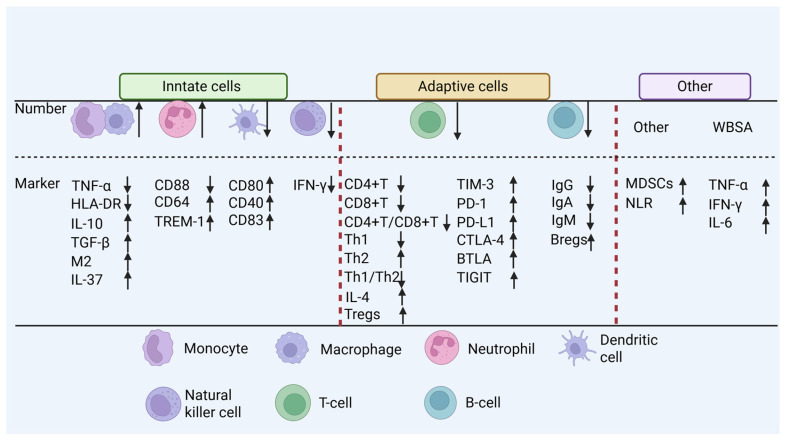
**Immune status monitoring.** TNF-α, tumor necrosis factor-alpha; HLA-DR, human leukocyte antigen-DR isotype; IL, interleukin; TGF-β, transforming growth factor-β; M2, M2 macrophage; CD, cluster of differentiation; TREM-1, triggering receptor expression on myeloid cells-1; IFN-γ, interferon-γ; Th1, T helper 1 cell; Th2, T helper 2 cell; Ig, immunoglobulin; Treg, T regulatory cell; Breg, B regulatory cell; TIM-3, T cell immunoglobulin and mucin domain-3; DP-1, programmed death-1; PD-L1, programmed death ligand-1; CTLA-4, cytotoxic T-lymphocyte associated protein-4; BTLA, B and T lymphocyte attenuator; TIGIT, T cell immunoreceptor with Ig and ITIM domains; MDSCs, myeloid-derived suppressor cells; NLR, neutrophil-to-lymphocyte ratio; WBSA, whole blood stimulation assay. The arrows denote changes in immune cell numbers and the levels of secreted or expressed cytokines/biomarkers under septic immunosuppression, with upward and downward directions representing increase and decrease, respectively. The dashed lines function solely as visual separators.

Monitoring immune status is a cornerstone of effective sepsis management; unfortunately, no single biomarker capable of definitively diagnosing sepsis or immunosuppression has been identified. This study focuses on the precise identification of the immunosuppressive state in sepsis through functional immune monitoring, biomarker profiling, and clinical recognition and phenotyping, aiming to improve diagnostic accuracy for septic patients with immunosuppression (Figure 4).

### 3.1. Monitoring the Immune Status in Sepsis

#### 3.1.1. Functional Monitoring of the Innate Immune System

##### Functional Monitoring of Monocytes

One hallmark of sepsis-induced immunosuppression is the functional impairment of monocytes/macrophages and their differentiation into hypoactive subsets [17]. These subsets exhibit diminished responses to stimuli such as LPS, characterized by reduced release of proinflammatory cytokines such as TNF-α and increased secretion of anti-inflammatory mediators such as IL-10 and TGF-β [9]. For example, Reyes et al. [72] employed single-cell RNA sequencing to identify an immunosuppressive monocyte subpopulation (designated the MS1 state) originating from aberrant hematopoiesis in the bone marrow of septic patients. MS1 cells display blunted responses to LPS stimulation, decreased TNF-α secretion, and altered expression of mitochondrial respiration-related genes; these features correlated with disease severity, as reflected by SOFA scores. Therefore, the emergence of the MS1 subset represents a measurable manifestation of immunosuppression in sepsis. However, its clinical translation is limited by the complexity, high cost, and lengthy turnaround time of single-cell sequencing, which precludes its use for real-time decision-making.

Notably, a significant decrease in the expression of mHLA-DR serves as a classical marker of immunosuppression [73,74]. Reduced mHLA-DR levels are associated with an increased risk of secondary infections, prolonged ICU and hospital stays, and increased mortality. Therefore, mHLA-DR represents a well-established and representative indicator for assessing immune status in septic patients. Nevertheless, its routine clinical application is challenged by the lack of standardized assays across centers and limited availability due to dependency on flow cytometry, which requires specialized equipment and personnel, often resulting in delays that hinder timely therapeutic guidance.

##### Functional Monitoring of Neutrophils

Neutrophils exhibit impaired chemotaxis, migration, and oxidative burst capacity during sepsis, leading to compromised pathogen clearance [23]. After migrating to sites of infection and engaging in bacterial phagocytosis and killing, neutrophils upregulate surface proteins such as CD64 [75] and triggering receptor expressed on myeloid cells-1 (TREM-1). Additionally, CD88 serves as another marker of C5a-mediated neutrophil dysfunction. Excessive C5a release suppresses CD88 expression, and reduced CD88 levels on neutrophils are closely associated with an increased risk of subsequent secondary infections [46]. In summary, the aforementioned markers and the impaired chemotactic capacity of neutrophils are strong predictors of immunosuppression. While their clinical adoption is hindered by the complexity of functional assays (e.g., chemotaxis) and a lack of standardized, validated clinical thresholds for surface markers like CD64, TREM-1, and CD88.

##### Functional Monitoring of DCs

In infectious diseases, DCs primarily perform their immune functions by activating effector T cells and regulating immune responses [76]. During the immunosuppressive phase of sepsis, the emergence of the mregDC subpopulation is often accompanied by high expression of maturation markers (CD80, CD40, and CD83) and immunoregulatory molecules (PD-L1, CD200, and Fas) [26]. Furthermore, the significant reduction in the number of DCs and their impaired function are closely associated with secondary infections and mortality [3,9,25]. However, the clinical translation of these findings faces significant challenges. The rare populations of mregDCs subsets, relies on advanced multicolor flow cytometry, a technique requiring specialized expertise and equipment that limits its availability in routine clinical practice.

##### Functional Monitoring of NK Cells

As pivotal effector cells, the functional status of NK cells is closely associated with clinical outcomes in sepsis patients [46]. NK cells exert their cytotoxic effects through the production of various cytokines, most notably IFN-γ [25]. Studies have demonstrated a dynamic inverse correlation between elevated TIM-3 expression and reduced IFN-γ production in NK cells in murine sepsis models [77]. Therefore, the serum IFN-γ concentration may serve as a useful indicator of the functional capacity of NK cells. However, its interpretation as a specific marker of NK cell function is complicated by its multiple cellular sources. Moreover, translating research markers like TIM-3 into clinical practice requires standardized assays and validated thresholds, which are currently lacking.

#### 3.1.2. Functional Monitoring of the Adaptive Immune System

##### Functional Monitoring of T Lymphocytes

Increased lymphocyte apoptosis, decreased proliferative capacity, T cell anergy, and shifts in lymphocyte subset proportions are characteristic features of immunosuppression or immunoparalysis in sepsis [78]. Among septic patients, 74% develop lymphopenia (absolute lymphocyte count, ALC < 1.0 × 10^9^/L) within 1–2 days of onset. Lower lymphocyte counts correlate with more severe organ dysfunction and higher 28-day and one-year mortality. An ALC persistently less than 760/μL for more than three days serves as an independent predictor of 28-day mortality in nonviral sepsis patients [36]. Therefore, lymphocyte counts provide a valuable reflection of immune status in sepsis.

A retrospective observational study further indicated that an elevated neutrophil-to-lymphocyte ratio (NLR) is associated with disease severity and represents an independent predictor of 28-day mortality [79]. Lymphocyte apoptosis leads to a marked reduction in CD4+ T cells, although changes in CD8+ T cell counts are more variable. In addition, lymphocyte apoptosis promotes a shift toward Th2-type immunity while suppressing Th1 responses, resulting in immune skewing [38]. Therefore, assessing the CD4+/CD8+ T cell ratio and the Th1/Th2 balance can offer insights into the immune status of a patient. For example, a decreased CD4+/CD8+ ratio suggests impaired adaptive immunity [46]. Notably, the number and proportion of Tregs are critical parameters for evaluating immune status in sepsis [14,46]. From a clinical feasibility perspective, the ALC and NLR have the distinct advantage of being readily available from routine complete blood count (CBC) analysis, offering rapid, low-cost results. In contrast, the detailed assessment of lymphocyte subsets (e.g., CD4+/CD8+ ratio, Tregs, Th1/Th2 balance) requires specialized flow cytometry, which is more complex, costly, and time-consuming, limiting its routine use outside of specialized centers.

##### Functional Monitoring of B Lymphocytes

Changes in B-cell subsets and levels of B-cell survival factors as well as alterations in the expression of apoptosis-related genes in B cells can collectively reflect their functional status. During the immunosuppressive phase of sepsis, although the total number of B cells decreases and their function is impaired, manifested as reduced antibody production, particularly that of IgM, their proportion among lymphocytes may increase [37]. Therefore, dynamically monitoring the proportional changes in B cells within the lymphocyte population may serve as a potential method for assessing immune status. Notably, serum concentrations of IgG, IgA, and IgM are commonly used as specific parameters that directly reflect the B-cell functional capacity [25].

Additionally, Muszynski et al. [80] reported that the ability of immune cells in whole blood to produce TNF-α upon LPS stimulation outperformed conventional monocyte counts in predicting multiple organ dysfunction syndrome (MODS). Similarly, compared with traditional lymphocyte counts, the ability of whole blood cells to produce IFN-γ following phytohemagglutinin (PHA) stimulation showed superior predictive value for MODS. Furthermore, emerging techniques such as the ELLA microfluidic immunoassay platform and enzyme-linked immunospot (ELISpot) assays enable the evaluation of both innate and adaptive immune function by measuring the production of cytokines (e.g., TNF, IFN-γ, and IL-6) in ex vivo-stimulated whole blood from healthy individuals or patients [81]. Clinically, serum immunoglobulin levels (IgG, IgA, IgM) are the most readily applicable B-cell function markers due to standardized laboratory testing. In contrast, monitoring B-cell proportion shifts requires flow cytometry. In addition, ELLA, ELISpot are complex, specialized, and lack standardization, which currently confines them primarily to research settings.

### 3.2. Biomarker Profiles

During sepsis, the profiles of host biomarkers and cytokines change dynamically. Therefore, biomarker-guided treatment stratification represents a promising strategy [54].

In the early hyperinflammatory phase, immune activation predominates, manifesting as a “cytokine storm”, complement activation, endothelial injury, coagulation abnormalities, and macrophage activation-like syndrome (MALS) [11]. The cytokine profile in this stage is characterized primarily by elevated levels of proinflammatory mediators such as TNF-α, IL-1β, IL-6 [78], and IL-3 [82]. Complement activation is reflected by increased C3a and C5a levels, whereas coagulation dysregulation involves tissue factor (TF), thrombin, and dysregulation of the protein C system. Endothelial dysfunction is indicated by elevated angiopoietin-2 levels, decreased sphingosine-1-phosphate (S1P) levels, and loss of barrier integrity [14].

In contrast, the late phase is predominantly immunosuppressive, featuring elevated expression of inhibitory receptors such as TIM-3 [45], PD-1, PD-L1, CTLA-4, and B and T lymphocyte attenuator (BTLA); a reduced antigen-presenting capacity of monocytes (e.g., mHLA-DR) and DCs [14]; and significantly increased levels of anti-inflammatory cytokines, including IL-4, IL-10, IL-37 [46], and TGF-β [9]. Notably, the expansion of MDSCs also serves as a key indicator of immunosuppression [31,32]. Furthermore, transcriptomic technologies enabling the identification of immunosuppression-related gene expression signatures [83], as well as recently proposed gene panels [84], offer additional tools for assessing immune status in septic patients.

### 3.3. Clinical Identification and Phenotyping

Sepsis is a highly heterogeneous syndrome, and its clinical management must move beyond a one-size-fits-all approach. An effective reduction in mortality requires stratification of patients on the basis of their immune status to guide targeted therapy, and deciding whether to enhance or suppress immune responses is essential to effectively reduce mortality [78]. Although conventional methods such as clinical presentation, immune cell functional assays, and cytokine profiling can provide a general assessment of immune status, they often lack the precision needed to accurately identify immunosuppressed patients or those at high risk of death.

The precise subtyping of sepsis patients using immunological assays and multiomics technologies, such as transcriptomics, proteomics, and metabolomics, represents a promising future direction. Current subtyping approaches are largely based on the immune response status of the patient [85,86] and levels of inflammation [87]. Although phenotyping holds considerable promise, it faces challenges, including technical complexity, high costs, a lack of standardized criteria and consensus, and difficulties in clinical implementation [78].

Therefore, rapid, bedside-compatible detection tools are better suited for clinical application. For example, the FilmArray^®^ platform (bioMérieux, Marcy-l’Étoile, France) can be used to construct a clinical worsening model that predicts death or hospital-acquired infection (HAI), as well as a model for identifying immunosuppression (e.g., mHLA-DR < 8000 molecules/cell). This enables the rapid identification of septic patients with immunosuppression, thereby guiding immunotherapy, facilitating clinical trial stratification, and allowing for dynamic monitoring of immune status [88].

## 4. Bedside: From Mechanisms to Treatment, Exploring Immunostimulatory Strategies

**Figure 5:**
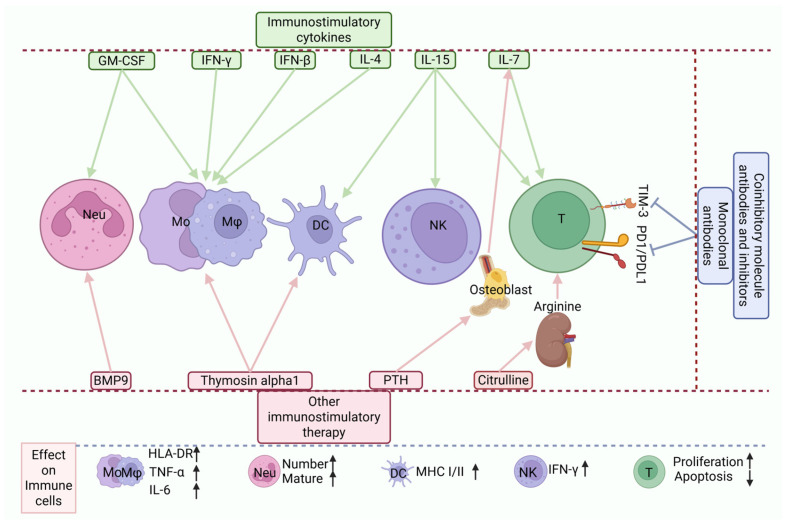
**Immunostimulatory therapy.** IFN-γ increases mHLA-DR expression on monocytes; GM-CSF increases mHLA-DR expression on monocytes and promotes the maturation of both monocytes and neutrophils; IFN-β reactivates the IFN-I pathway, promotes monocyte maturation, and restores the production of TNF and IL-6; IL-4 promotes immune cells, increases the immunoreactivity of monocytes, and shifts the metabolic mode of anergic immune cells from glycolysis to oxidative phosphorylation; IL-7 inhibits T cell apoptosis and promotes T cell proliferation and functional recovery; IL-15 stimulates the proliferation and activation of CD8+ T cells, NK cells, and DCs and promotes IFN-γ secretion by NK cells; and immune checkpoint blockade therapy uses monoclonal antibodies to block TIM-3, and the PD-1/PD-L1 pathway can stimulate T cell proliferation, restore T cell function and inhibit their apoptosis. Other agents and mechanisms: BMP9 activates monocyte function and enhances the ability of monocytes to combat bacteria; thymosin-α1 stimulates TLR2 and TLR9, enhances the antigen-presenting capacity of DCs, promotes the production of IL-2, IL-12, IFN-γ, and IFN-α by immune cells, and increases mHLA-DR expression; PTH restores osteoblast function, thereby increasing IL-7 production and promoting T cell proliferation and function; Citrulline is metabolized in the kidneys to produce arginine, which improves mitochondrial function in T cells, enhances T cell responsiveness, and simultaneously inhibits the proliferation of immunosuppressive cells. IFN-β, interferon-β; IFN-I, interferons; IL, interleukin; GM-CSF, granulocyte–macrophage colony-stimulating factor; TIM-3, T cell immunoglobulin and mucin domain-3; PD-1, programmed death-1; PD-L1, programmed death ligand-1; BMP9, bone morphogenetic protein 9; thymosin-α1, thymosin-alpha-1; PTH, parathyroid hormone; mHLA-DR, monocytic human leukocyte antigen-DR isotype; TNF-α, tumor necrosis factor-α; TLR, Toll-like receptor; MHC, major histocompatibility complex. Green arrows denote cytokine therapies and their respective target cells, signifying a promotional effect. Pink arrows represent other classes of immunostimulatory therapies and their targets, similarly signifying promotion. Blue arrows signify the promotion of target cell proliferation and functional recovery via immune checkpoint blockade. Black arrows indicate the consequent functional alterations in immune cells upon targeting by immunostimulatory therapies, with an upward direction denoting improvement.

The management of sepsis is a protracted process that encompasses early recognition and initial standardized interventions, such as antibiotic administration, fluid resuscitation, and vasoactive drug support. In addition, early mobilization has been shown to mitigate muscle atrophy, shorten ICU stays, and improve functional recovery, thereby reducing and preventing complications, including immunosuppression [12]. Following the detection of immunosuppression, selecting appropriate immune reconstitution therapies may increase long-term survival in septic patients. This section discusses therapeutic approaches for sepsis-induced immunosuppression in the context of underlying mechanisms, with the aim of providing insights for clinical practitioners (Figure 5).

### 4.1. Therapies Targeting the Innate Immune System

#### 4.1.1. IFN-γ

IFN-γ plays a dual role in immune regulation during sepsis. In the early hyperinflammatory phase, NKT cells promote IFN-γ production by NK cells via the mTORC1 signaling pathway, which suppresses macrophage phagocytic function and contributes to the development of immunosuppression. Rapamycin, an mTOR pathway inhibitor, has been shown in mouse models of sepsis to improve macrophage phagocytosis and reverse immunosuppression when it is administered early in sepsis [89].

In contrast, during the immunosuppressive phase, IFN-γ can help restore mHLA-DR expression and enhance cytokine secretion capacity. Small-scale clinical studies have indicated potential benefits from IFN-γ administration in this setting [43,83,90]. Cheng et al. [51] reported that IFN-γ restores lactate production in immunotolerant monocytes and enhances mTOR pathway activity. In a clinical pilot study involving patients with fungal sepsis, the immunometabolic defects in humans were partially restored by therapy with recombinant IFN-γ, suggesting that IFN-γ may serve as a therapeutic strategy to restore immunometabolic function.

#### 4.1.2. GM-CSF

Granulocyte–macrophage colony-stimulating factor (GM-CSF) enhances the production, maturation, and function of monocytes, macrophages, and neutrophils and may be beneficial in patients with positive immunosuppression markers such as low HLA-DR expression [91]. Previous clinical trials have demonstrated that GM-CSF can increase mHLA-DR levels in immunosuppressed sepsis patients [3,92]. For example, a multicenter randomized clinical trial revealed that GM-CSF elevated mHLA-DR expression and neutrophil counts, but no significant effect on the prevention of ICU-acquired infection in sepsis immunosuppression. So its clinical benefits still require validation through larger studies [93].

#### 4.1.3. IL-4

Interleukin-4 (IL-4) has traditionally been regarded as an anti-inflammatory cytokine that inhibits proinflammatory factors such as TNF and IL-6. However, recent studies have revealed that in addition to its acute anti-inflammatory effects mediated by the signal transducer and activator of transcription 6 (STAT6) pathway, IL-4 can also induce trained immunity through the phosphoinositide 3-kinases-mammalian target of rapamycin (PI3K–mTOR) pathway, thereby enhancing the long-term immune responsiveness of human monocytes and reversing immune tolerance induced by LPS in septic mice. Furthermore, IL-4-induced trained immunity is accompanied by metabolic reprogramming that strengthens both glycolysis and OXPHOS, as well as epigenetic remodeling [94].

### 4.2. Therapies Targeting the Adaptive Immune System

#### 4.2.1. IL-7

Interleukin-7 (IL-7) plays a critical role in promoting T cell proliferation, preventing apoptosis, and enhancing the recovery of T cell numbers and function. IL-7 is a key cytokine for early lymphocyte development [95] and is currently the only agent in clinical development proven to rapidly, sustainably, and safely reverse severe lymphopenia. The recommendation is that recombinant human IL-7 (rhIL-7) be initiated within 48 h after severe lymphopenia is confirmed (absolute lymphocyte count, ALC < 500/μL) [36]. A prospective study demonstrated that treatment with rhIL-7 increases the absolute lymphocyte count in severe COVID-19 patients with lymphopenia and reduces the incidence of late-onset nosocomial infections, without significant adverse effects, ultimately lowering the late-stage morbidity and mortality of COVID-19 [96]. Daix [97] et al. also found in a clinical trial that IL-7 therapy can reverse severe lymphopenia in sepsis. Furthermore, Marton [98] et al. reported that IL-7 significantly improves in vitro T cell function in patients with immunosenescence. Some studies have revealed that during sepsis, osteoblasts are rapidly impaired, and the number of common lymphoid progenitors (CLPs) in the bone marrow decreases significantly. This is partly attributed to elevated levels of granulocyte colony-stimulating factor (G-CSF), a major driver of osteoblast reduction. Although osteoblasts are important sources of IL-7, their loss helps explain why lymphocyte counts remain low even after the peak of apoptosis has passed. Therefore, neutralizing G-CSF or using parathyroid hormone (PTH) to stimulate IL-7 secretion by osteoblasts represents a potential strategy to reverse immunosuppression and improve the survival rate of septic mice [99]. However, optimal therapeutic strategies and long-term effects of IL-7 in sepsis treatment remain to be further investigated.

#### 4.2.2. IL-15

Interleukin-15 (IL-15) has been shown to stimulate the proliferation and activation of CD4+/CD8+ T cells, NK cells, and DCs [95]. In models of septic mice, IL-15 therapy improved survival by counteracting apoptosis and immunosuppression and preventing cell death in immune cell populations and the gut epithelium. Moreover, IL-15 therapy enhanced immune function by increasing both the level of circulating IFN-γ and the frequency of IFN-γ-producing NK cells. Collectively, these findings identify IL-15 as a potential novel treatment for sepsis [100]. However, the role of IL-15 in immunomodulation for sepsis warrants further investigation.

### 4.3. Therapies Targeting Immune Checkpoints

In recent years, immune checkpoints, such as PD-1, PD-L1 and TIM-3, have attracted significant attention in sepsis-associated immunosuppression. Sustained and increased expression of PD-1 on T cells, monocytes, and granulocytes, along with increased PD-L1 expression on antigen-presenting cells, is recognized as a hallmark of immunosuppression [78]. Monoclonal antibodies targeting PD-1 or its ligands have been shown to inhibit these coinhibitory signals at the T cell–APC interface, preserve T cell function, and exert antiapoptotic effects [101]. A preclinical study revealed that mice in which the PD-1/PD-L1 interaction was inhibited had better survival rates after sepsis was induced, indicating that blocking these immune checkpoints is a potential treatment strategy for sepsis [102]. A study by Lee [103] et al. demonstrated that combination therapy with IL-6 and PD-1 antibody blockade improves hematological parameters and bacterial clearance, reduces organ neutrophil infiltration, decreases splenic T lymphocyte apoptosis and ameliorates histopathological damage. Separately, research by Gossez [104] et al. showed that following plasma cell clearance with bortezomib, the decreased PD-L1 expression inhibited PD-1/PD-L1 binding, resulting in restored T cell proliferation and ameliorated weight loss in septic mice. Moreover, blocking the TIM-3/HMGB1 pathway has been proven to alleviate sepsis-induced immunosuppression and improve survival in mice [45]. In summary, therapies targeting these immune checkpoints represent a promising future direction for reversing immune cell exhaustion in sepsis.

### 4.4. Other Immunomodulatory Strategies

#### 4.4.1. Thymus-Replacement Therapy

Sepsis-induced thymic involution or atrophy is closely associated with T cell aging and impaired adaptive immune responses [105]. Sommer et al. [106] reported that reduced thymic output accelerates lymphocyte aging and ultimately contributes to lymphopenia. Thymosin α1, an endogenous 28-amino-acid peptide that is primarily synthesized in the thymus, exhibits multiple immunomodulatory functions: (1) potential restoration of immune responses in septic patients; (2) stimulation of Toll-like receptor-2 (TLR2) and TLR9 on bone marrow and DCs, thus enhancing MHC class I/II expression and promoting cytokine production (e.g., IL-2, IL-12, IFN-γ, and IFN-α) by immune cells; and (3) activation of complement receptor-mediated phagocytosis to support pathogen clearance, along with the enhancement of cytotoxic T- and NK-cell responses [43].

Wu et al. [107] reported that adjunctive therapy with thymosin α1 significantly increased mHLA-DR expression and improved 28-day survival in patients with severe sepsis. Therefore, thymus replacement therapy represents a promising approach for managing immunosuppression in sepsis.

#### 4.4.2. Immunonutrition Supplementation

T cell dysfunction and the expansion of immunoregulatory cells, including Tregs and MDSCs, are hallmarks of immunosuppression in sepsis. Arginine deficiency serves as a key trigger, particularly because of high arginase-1 expression in MDSCs, which depletes extracellular arginine pools. As a precursor of arginine, citrulline bypasses hepatic metabolism and is efficiently converted into arginine in the kidneys. This helps restore systemic arginine levels, improves T cell mitochondrial function, enhances immune reactivity, suppresses the expansion of immunosuppressive cells, and reduces the risk of lung injury and secondary infection, but did not significantly improve the overall survival of mice. Therefore, citrulline supplementation is a potential nutritional intervention strategy for patients in the immunosuppressive phase of sepsis [108].

Omega-3 polyunsaturated fatty acids exert multilevel anti-inflammatory and proresolving effects by competitively inhibiting proinflammatory fatty acid metabolism, generating metabolites with low inflammatory activity, and synthesizing specialized proresolving mediators that actively promote inflammation resolution [109]. Singer et al. [110] reported that low-dose, continuous enteral administration of omega-3 fatty acids improves respiratory function, oxygenation, and nutritional status in patients with acute lung injury (ALI) while shortening the duration of mechanical ventilation and the ICU stay. Recent studies further revealed a significant inverse correlation between baseline omega-3 levels and the risk of hospitalization for sepsis, suggesting that omega-3 supplementation may serve as a preventive strategy against sepsis development [111]. This could represent a potential approach to preventing secondary infections in post-discharge sepsis survivors with prior immunosuppression.

#### 4.4.3. Other Molecular-Targeted Approaches

Bone morphogenetic protein 9 (BMP9) activates macrophages via the ALK1/Smad1/5 signaling pathway, enhancing peritoneal macrophage infiltration and bacterial clearance capacity and thereby improving survival outcomes in murine models of sepsis-induced immunosuppression [112]. Additionally, IFN-β reactivates the IFN-I pathway, reverses the immunosuppressive phenotype, promotes monocyte maturation, and restores the production of inflammatory cytokines (e.g., TNF and IL-6), leading to functional immune recovery [18] (Table 1).

### 4.5. Current Challenges and Therapeutic Dilemmas

#### 4.5.1. The Need for Personalized Treatment

Research has indicated that although sepsis mortality has slightly declined over the past decade, the fatality rate of septic shock has not improved significantly since 2011, suggesting that current treatment strategies have reached a plateau. Moreover, despite variations across regions and study types, overall mortality remains high, underscoring the substantial room for improvement in sepsis management [113].

In terms of sepsis therapeutics, although numerous immunomodulatory approaches have been shown to be effective in animal studies, nearly all have failed in human clinical trials. A key reason is that sepsis is not a single disease; different subphenotypes exhibit significant heterogeneity in immune status, organ dysfunction, and treatment response [114]. Clinical practice tends to categorize patients with similar presentations together to facilitate early recognition and standardized treatment (e.g., antibiotics and fluid resuscitation), often overlooking underlying biological variability. This has hindered the development of targeted therapies.

Stratifying patients into distinct endotypes on the basis of biological mechanisms could help identify subgroups likely to respond to specific interventions, distinguish high-risk populations for closer monitoring, and enable focused intervention. However, such approaches require biomarker support and are highly complex to implement [115].

#### 4.5.2. Host-Directed Therapy (HDT)

HDT represents an emerging strategy that modulates host immune responses or factors to enhance the anti-infective capacity, control inflammation, and inhibit pathogen replication or persistent infection. HDT has shown promise in the treatment of various bacterial and viral infections. The core concept lies not in directly targeting pathogens but in regulating host responses to achieve multiple effects, including anti-infection, anti-inflammatory, and immune-enhancing outcomes. In the future, HDT is expected to become an integral component of personalized anti-infective therapy, complementing existing antimicrobial drugs and ushering in a new era of “precision + immunomodulatory” anti-infective treatment [116].

#### 4.5.3. Timing and Duration of Therapy

As previously discussed, the hyperinflammatory and immunosuppressive phases in sepsis are not mutually exclusive but often coexist from the early stages of the disease, with one state predominating at different time points [91]. Given that the patient’s immune status is highly dynamic, immunomodulatory interventions must be carefully timed. For example, during the hyperinflammatory phase, targeted anti-inflammatory strategies may be appropriate, whereas in the immunosuppressive phase, immune-reconstituting therapies are needed. This approach necessitates close monitoring of the immune status in septic patients to avoid reigniting excessive inflammation during immune restoration efforts.

## 5. Conclusions and Future Perspectives

### 5.1. Future Directions

The management of sepsis requires multidisciplinary collaboration involving intensive care, infectious diseases, immunology, trauma surgery, and other specialties. Sepsis-induced immunosuppression is a critical determinant of patient outcomes. Its onset and duration vary among individuals, and although great progress has been made in understanding its underlying mechanisms in recent years, translating these discoveries into effective therapies remains a considerable challenge. Greater in-depth and comprehensive exploration of the immunopathological mechanisms of sepsis is still needed.

Future research should focus on the following priorities:

- Utilizing multiomics technologies integrated with artificial intelligence (AI) for the precise and reliable subtyping of sepsis patients. This approach, which spans the genome, transcriptome, proteome, metabolome, and single-cell level, aims to achieve accurate diagnosis and facilitate personalized treatment for septic individuals. AI can integrate clinical data with multiomics data, identify nonlinear features, and accomplish patient stratification, immune status assessment, and disease prediction [117]. For example, Dalal et al. [118] proposed an AI model that combines deep learning for time series data with conformal prediction for the early prediction of sepsis in hospitalized non-ICU patients. Furthermore, Qin et al. [119] employed machine learning for the early phenotypic identification of pediatric sepsis, discerning a PedSep-D phenotype characterized by high mortality and distinct immune abnormalities. Consequently, the combined application of omics technologies and AI holds significant potential in the diagnosis and treatment of sepsis.
- Developing rapid, point-of-care immune function assays: Creating tools for real-time, dynamic immune monitoring at the bedside, such as the ELLA platform [81] and FilmArray^®^ system [88], to closely track changes in the immune status of patients is necessary.
- Exploration of combination therapies targeting multiple immune defects: Developing and evaluating combination immunomodulatory strategies tailored to the specific immune deficits of individual sepsis patients is essential. Examples include IL-7 combined with immune checkpoint inhibitors to simultaneously promote T cell proliferation and prevent exhaustion or IL-7 paired with β-blockers to support immune reconstitution while modulating excessive inflammation [36].
- Follow-up system: Establishing a standardized follow-up system is crucial, as studies have shown that professional rehabilitation guidance can effectively improve the long-term survival rates of patients. For example, the sepsis transition and recovery (STAR) program, led by Taylor et al. [120], significantly reduced the 90-day mortality rate in sepsis patients. Therefore, implementing a standardized postdischarge follow-up system that includes rehabilitation guidance, chronic disease management, and psychological support is essential for improving the long-term outcomes of sepsis survivors.

### 5.2. Conclusions

Sepsis-induced immunosuppression is a pivotal determinant of patient outcomes and arises from a complex interplay of immune dysfunction, metabolic reprogramming, and epigenetic alterations. The lack of standardized, rapid bedside diagnostics impedes precise intervention, while the failure of universal therapies highlights the necessity for patient stratification on the basis of immune phenotypes. Future efforts should integrate multiomics and AI for precise endotyping and dynamic monitoring. Promising strategies such as IL-7, GM-CSF, and immune checkpoint inhibitors require validation in tailored trials, with combination therapies offering significant potential. Ultimately, overcoming sepsis demands a comprehensive “bench-to-bedside” approach that strengthens primary care detection, implements personalized immunotherapy, and establishes systematic follow-up. Only through such an integrated, precise framework can we improve survival and quality of life for sepsis survivors.

## Figures and Tables

**Table 1:** Key immunomodulatory agents in the therapy of sepsis-induced immunosuppression.

Immunomodulatory Agent	Target/Agent	Mechanism	Clinical Trial Status/Key Outcomes (with Citation)	Limitations
IFN-γ	Monocytes	Enhances mTOR pathway activity	Clinical trial/partially restore the immunometabolic defects [51]	Limited sample size, lack of a control group, only partially restored glycolysis, unclear correlation between metabolic indicators and clinical outcomes, potential pro-inflammatory risks, insufficient evaluation of metabolic side effects
IFN-β	Monocyte	Reactivates the IFN-I pathway	Restored IFN- I responses and proinflammatory cytokine production and induced monocyte maturation in vitro [18]	The absence of in vivo therapeutic validation of IFN-β, no testing in animal models or clinical patients to verify its in vivo efficacy
IL-4	Monocytes	PI3K–mTOR pathway	Enhancing the long-term immune responsiveness of human monocytes, reversing immune tolerance induced by LPS in septic mice [94]	LPS cannot fully replicate the complex pathophysiological state seen in real sepsis patients (such as multi-organ failure, pathogen diversity, etc.), and large-scale animal models or early-stage clinical trials are still required.
GM-CSF	Monocytes, macrophages, and neutrophils	Increased mHLA-DR levels	Increased mHLA-DR levels, but no effect on the prevention of ICU-acquired infection in sepsis immunosuppression [93]	Difficulty in patient enrollment, insufficient sample size, and premature termination of the study.
IL-15	CD4+/CD8+ T cells, NK cells, and DCs	Stimulate the proliferation and activation of immune cells and increase the IFN-γ level	Increase the IFN-γ level and frequency of IFN-γ-producing NK cells [100]. To our knowledge, no clinical trials have been reported	No testing in clinical patients to verify its in vivo efficacy.
IL-7	T cell Thymus	Recovery of T-cell numbers and function	Lowering the late-stage morbidity and mortality of COVID-19 [96]. Reverse severe lymphopenia in sepsis, but no significant differences between the two groups in terms of mortality, secondary infection rates, ICU length of stay [97]. Promotes the maturation of T cells in the thymus [106]	The small study sample size and early termination of enrollment reduced statistical power, potentially masking the drug’s true effects on certain clinical endpoints (such as mortality and hospital length of stay)
TIM-3 inhibitor	T cell	Recovery of T-cell numbers and function	Alleviate sepsis-induced immunosuppression and improve survival in mice [45]	No testing in clinical patients to verify its in vivo efficacy in sepsis.
PD1/PDL-1 inhibitor	T cell	Recovery of T-cell numbers and function	Improves hematological parameters and bacterial clearance, reduces organ neutrophil infiltration, decreases splenic T lymphocyte apoptosis and ameliorates histopathological damage [103]. restored T cell proliferation and ameliorated weight loss in septic mice [103]	No testing in clinical patients to verify its in vivo efficacy in sepsis.
BMP9	Macrophages	ALK1/Smad1/5 signaling	Improve survival outcomes in murine models of sepsis-induced immunosuppression [112]	No testing in clinical patients to verify its in vivo efficacy in sepsis.
PTH	Osteoblasts	stimulate IL-7 secret	Improve the survival rate of septic mice [99]	No testing in clinical patients to verify its in vivo efficacy in sepsis.
Citrulline	T cell	Converted into arginine in the kidneys	Increases blood arginine levels after renal metabolism, improves T-cell function, suppresses immunosuppressive cell expansion, and mitigates the severity of lung injury and secondary infections, but it does not significantly improve overall survival [108]	No testing in clinical patients to verify its in vivo efficacy in sepsis.

## Data Availability

No new data were created or analyzed in this study. Data sharing is not applicable to this article.
